# Complete pathological regression of hepatocellular carcinoma with portal vein thrombosis treated with sorafenib

**DOI:** 10.1186/1477-7819-11-171

**Published:** 2013-08-02

**Authors:** Sabrina Kermiche-Rahali, Aude Di Fiore, Fanny Drieux, Frédéric Di Fiore, Arnaud François, Michel Scotté

**Affiliations:** 1 Department of Digestive Surgery, Rouen University Hospital, 1 Rue de Germont, 76031 Rouen, France; 2 Department of Hepatology and Gastroenterology, Rouen University Hospital, 1 Rue de Germont, 76031 Rouen, France; 3 Department of Pathology, Rouen University Hospital, 1 Rue de Germont, 76031 Rouen, France; 4 INSERM U1073, Rouen University Hospital, 1 Rue de Germont, 76031 Rouen, France

**Keywords:** Hepatocellular carcinoma, Sorafenib, Liver resection

## Abstract

Sorafenib is a molecular-targeted therapy used in palliative treatment of advanced hepatocellular carcinoma (HCC) in Child-Pugh A patients. We describe the case of a patient who presented with a large HCC in the left liver associated with portal vein thrombosis (PVT). After 9 months of sorafenib treatment, reassessment showed that the tumors had decreased in size with recanalization of the portal vein. A lateral left hepatectomy was performed and pathology showed complete necrosis of the tumor. Sorafenib can downstage HCC in patients with cirrhosis allowing further surgical resection.

## Background

Hepatocellular carcinoma (HCC) is the most common type of primary liver tumor and is the fifth most common malignancy worldwide [1]. Variables related to early HCC status (single tumor ≤5 cm or three nodules ≤3 cm), the so-called Milan criteria, define good prognosis and are accessible to curative therapies, such as surgical resection, liver transplantation and percutaneous ablation [2,3]. Surgical resection is still considered the most effective treatment for HCC. However, only 30% of all patients are diagnosed at early stage and therefore able to benefit from curative therapies [2]. These patients achieve 5-year survival rates of 50% to 75% [3].

Most patients are diagnosed at advanced stage. The main prognostic factors are related to tumor status defined by the number and size of nodules, presence or absence of vascular invasion, presence or absence of extrahepatic spread, liver function (Child-Pugh class, serum bilirubin and albumin levels, and portal hypertension), and general health status defined by the Eastern Cooperative Oncology Group (ECOG) classification.

Untreated patients with advanced-stage disease, that is ECOG performance status grade 1 or grade 2, and/or vascular invasion or extrahepatic spread, have a median survival of 6 to 7 months [4].

Sorafenib (BAY 43–9006, Nexavar; Bayer, Leverkusen, Germany), a multi-target tyrosine kinase inhibitor used in palliative treatment of advanced HCC, can improve the survival of patients [5]. Nevertheless, Llovet *et al*. [5] demonstrated in a multicenter, phase III, double-blind, placebo-controlled trial that median time to symptomatic progression did not improve, and only 2% of patients achieved partial radiological response and none complete response. We report the case of a patient with locally advanced HCC associated with portal vein thrombosis (PVT). The patient achieved complete regression of HCC using sorafenib treatment allowing further curative surgical resection.

## Case presentation

**Figure 1:**
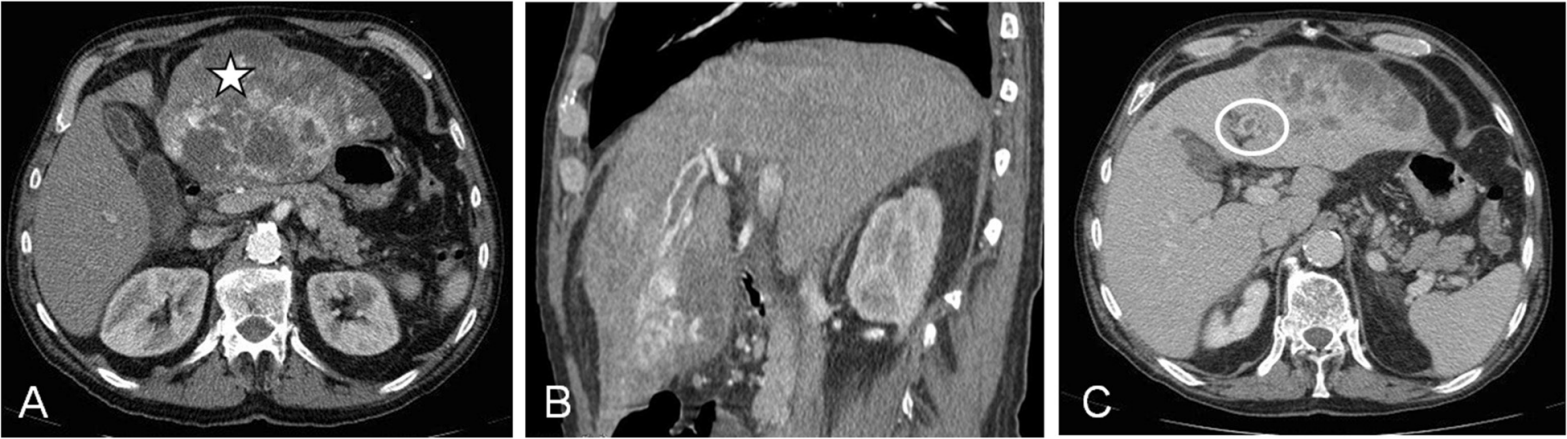
**Computerized tomography (CT) scan features of the liver tumor.** Liver tumor with **(A)** arterial enhancement (star), **(B)** portal washout and **(C)** portal vein thrombosis (PVT) (circle). CT, computerized tomography; PVT, portal vein thrombosis.

A 68-year-old man presented to Rouen University Hospital, Rouen, France, with asthenia, diffuse abdominal pain and cirrhosis. Hepatitis B and C virus serologic status were negative and iron status was normal. Cirrhosis was related to alcohol abuse. Abdominal ultrasound showed hepatomegaly and a liver tumor measuring 14 cm located in the left lobe. An abdominal computerized tomography (CT) scan confirmed a lesion in the left liver measuring 15.7 cm and with left intrahepatic portal branch thrombosis (Figure 1). Typical radiological features, including hypervascular and portal phase washout, together with an increase in alpha-fetoprotein (AFP) serum levels at 986 ng/mL (normal <20 ng/mL), confirmed diagnosis of HCC without performing liver biopsy [2]. The size of the lesion associated with a portal thrombus prohibited curative surgical treatment [3]. Considering the patient’s general state of health and the Child-Pugh class (A6), palliative treatment with sorafenib (800 mg/day) was initiated in January 2011.

After 3 months of treatment, evaluation showed normalization of AFP levels (from 986 ng/mL to 7 ng/mL and decrease in hepatic lesion size (from 15.7 cm to 7 cm). Side effects included grade 2 to 3 hand-foot syndrome and diarrhea. Due to this objective radiological response, sorafenib treatment was continued with dose adjustment to 600 mg/day.

**Figure 2:**
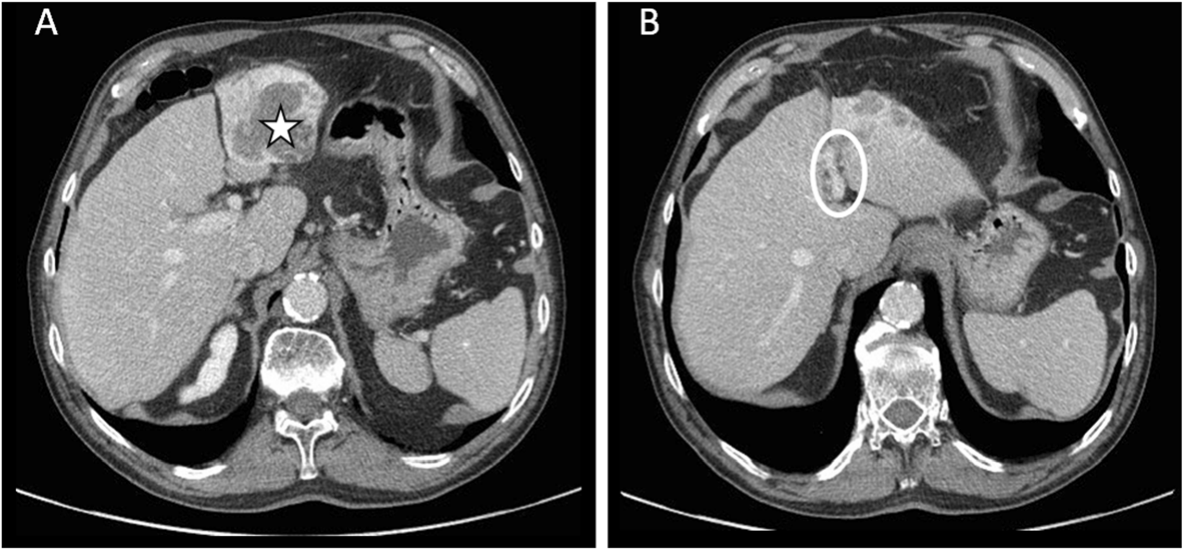
**Computerized tomography (CT) scan of the left liver tumor lesion after 9 months of treatment with sorafenib. (A)** Left liver tumor lesion (star), and **(B)** regression of the lesion with recanalization of the left portal branch (circle). CT, computerized tomography.

After 9 months of treatment, AFP levels decreased to 5.3 ng/mL, and CT scan showed 60% regression of the hepatic lesion measuring 6 cm long with recanalization of the left portal branch (Figure 2).

One month after stopping sorafenib, surgical treatment was proposed and a left liver lobectomy was performed without vascular clamping. There were no postsurgical complications and the patient was discharged from hospital 5 days after surgery.

**Figure 3:**
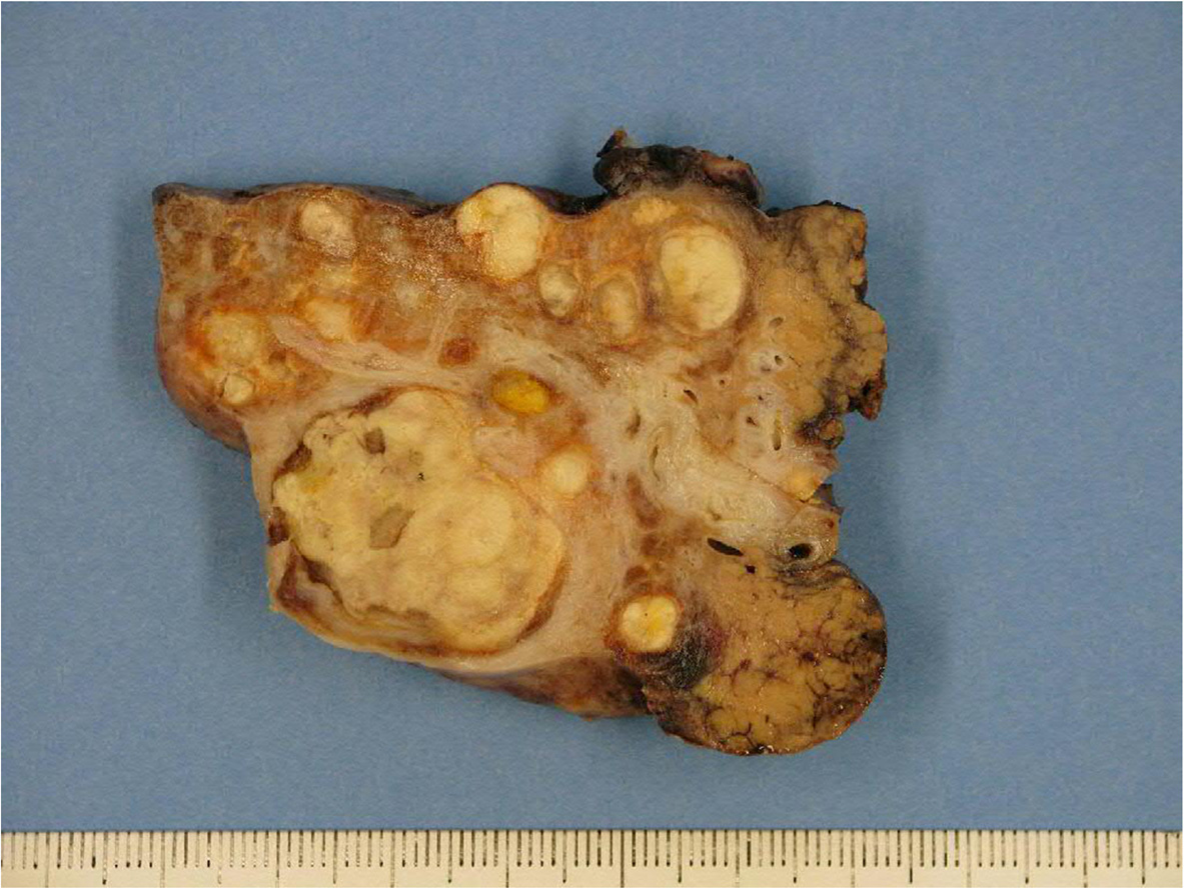
Gross examination of cirrhotic liver parenchyma with multiple white nodules.

**Figure 4:**
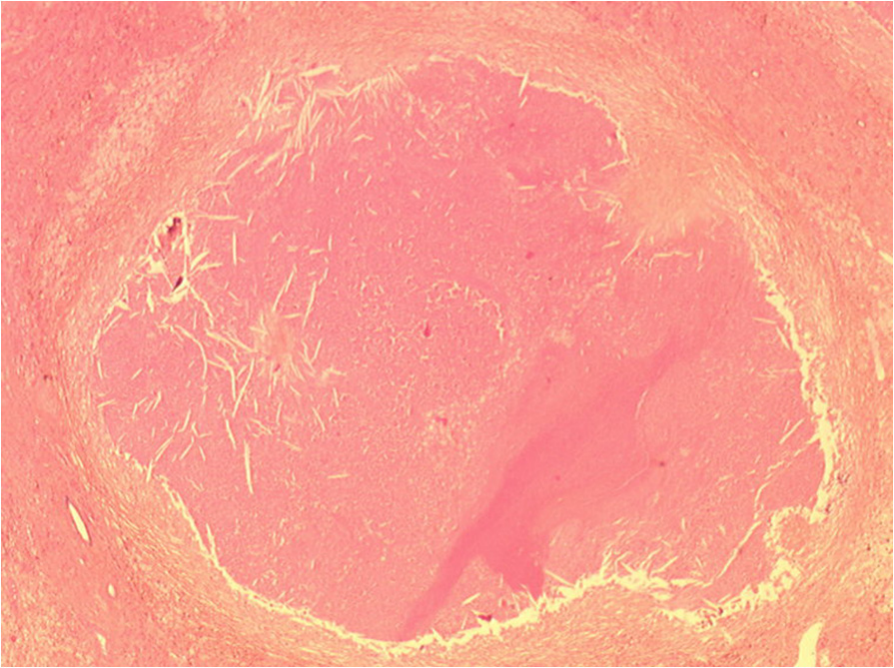
**Central eosinophilic amorphous necrosis surrounded by fibrous tissue without residual tumor cell.** Hematoxylin and eosin stain, × 25 magnification.

Gross pathological examination showed multiple white nodules and the whole lesion measured 6 cm × 4 cm × 5 cm. The nodules were well circumscribed by fibrous tissue. Microscopic examination revealed nodules with a central necrotic core, surrounded by a hyalinized fibrotic capsule. No residual tumor cell was observed. The surrounding liver parenchyma was cirrhotic (METAVIR F4) (Figures 3 and 4). The patient showed no recurrence of HCC 14 months after liver resection.

## Discussion

In this case, a patient with cirrhosis whose HCC was initially unresectable received sorafenib with palliative intent, allowing curative surgical resection. There was no evidence of residual tumor cell at microscopic examination. To our knowledge, only one case of complete pathological response induced by sorafenib treatment in a patient with cirrhosis and HCC has been reported in the literature [6].

Diagnostic criteria for HCC of a cirrhotic liver are mainly biological and radiological. Previously, an algorithm for investigation of liver nodules was proposed by the Barcelona Clinic Liver Cancer (BCLC) Group [7]. Diagnosis depends on the size and radiological characteristics of the nodule, associated with the rate of AFP and, in some cases, biopsy. Subsequently, the strategy for staging and treatment assignment for patients diagnosed with HCC was also proposed by the BCLC Group. In a recent retrospective observational study, Torzilli *et al*. [8] demonstrated that patients with advanced tumor can benefit from surgical resection and suggested that therapeutic guidelines should be updated.

HCC is a hypervascular tumor. Sorafenib is the only multi-kinase inhibitor that blocks receptor tyrosine kinases, such as vascular endothelial growth factor receptor (VEGFR) or platelet-derived growth factor receptor (PDGF-R), and also RAF serine/threonine kinases along the RAF/MEK/ERK pathway [9]. Thus, sorafenib targets both tumor cell proliferation and angiogenesis.

The efficacy of sorafenib in HCC has already been demonstrated in two pivotal, multicenter, phase III, placebo-controlled clinical trials. The SHARP Study Group [5] accrued more than 600 patients with advanced HCC and cirrhosis (Child-Pugh A) not previously treated with any systemic therapy. This study showed for the first time an advantage in terms of median overall survival, which was 10.7 months for sorafenib versus 7.9 months for placebo (*P* = 0.0006). However, median time to symptomatic progression did not differ significantly between the sorafenib and placebo group (4.1 months and 4.9 months, respectively; *P* = 0.77). In fact, only seven patients (2%) treated with sorafenib were considered responders according to Response Evaluation Criteria In Solid Tumors (RECIST) criteria. No complete response was recorded. Similarly, the second phase III clinical study, carried out in the Asia-Pacific region [10], recruited and randomized a total of 226 patients in a 2:1 fashion. Median overall survival of patients in the sorafenib group was 6.2 months, which was significantly better than the 4.1 months achieved in the placebo group (*P* = 0.0155). In the analysis of best response, five of 150 patients in the sorafenib group (3.3%) achieved partial response. There was also no complete response.

Despite the demonstrated effectiveness of sorafenib, there are few reported cases of resection in patients with biological or radiological response. Irtan *et al*. [11] reported two cases of downstaging of locally advanced HCC after sorafenib treatment, allowing curative resection with complete pathological response. However, in neither of these cases was the nontumoral liver cirrhotic. Both patients developed chronic liver disease (METAVIR F3) due to hemochromatosis and hepatitis B. The Barbier *et al*. study [12] described two other cases of surgery after sorafenib in two patients with chronic disease due to hepatitis C and chronic alcoholism, respectively. Nevertheless, histopathological examination found 35% and 60% of tumor necrosis, along with residual tumor cell, respectively. To our knowledge, only one case report of complete pathological regression in severe chronic liver disease (METAVIR F4) has been reported in the literature [6]. No tumor cells were found and recanalization of the portal vein was complete.

Recanalization of the portal vein is another interesting point. PVT is now considered as the main negative prognostic factor in patients with HCC. HCC with malignant PVT has poor prognosis, thus prohibiting curative approaches. In contrast, benign (fibrin clot) PVT is not a contraindication to curative treatment. Patients with cirrhosis and HCC may develop either benign or malignant PVT. In this context, critical evaluation of PVT by imaging techniques and diagnostic criteria [13] is mandatory to differentiate benign and malignant PVT [14]. In these settings, Li *et al*. [15] recently suggested that vascular endothelial growth factor may play a pivotal role in HCC angiogenesis, and in PVT onset and evolution. Sorafenib could exert a beneficial effect on PVT by inhibiting the VEGFR pathway.

## Conclusions

Sorafenib opens a new window for treatment of locally advanced HCC and highlights the importance of identifying factors that could be associated with good response to this therapy. However, complete tumor regression and necrosis increase difficulties in both tissue analysis and search for predictive factors of good response to the tumor (tumor phenotypes, biological markers, and so on). Furthermore, close monitoring and regular reassessment of patients is mandatory, in order to promptly identify good responders to sorafenib and subsequently propose surgical treatment or other curative treatment. Moreover, although a small number of hepatic resections following sorafenib administration have been reported, no intraoperative or postoperative complications related to preoperative treatment have been observed [16].

Therefore, our case report suggests that neoadjuvant sorafenib treatment could be a relay to surgery in selected patients.

## Consent

Written informed consent was obtained from the patient for publication of this case report and any accompanying images. A copy of the written consent is available for review by the Editor-in-Chief of this journal.

## Abbreviations

AFP: Alpha-fetoprotein; BCLC: Barcelona Clinic Liver Cancer; CT: Computerized tomography; ECOG: Eastern Cooperative Oncology Group; HCC: Hepatocellular carcinoma; PDGF-R: Platelet-derived growth factor receptor; PVT: Portal vein thrombosis; RECIST: Response Evaluation Criteria In Solid Tumors; VEGFR: Vascular endothelial growth factor receptor.

## Competing interests

The authors declare that they have no competing interests.

## Authors’ contributions

SKR and MS did operate on the patient, analysed and interpreted data and wrote the manuscript. AdF and FdF have treated the patient with sorafenib and participated to followup FD and AF performed the pathological analysis. All authors read and approved the final manuscript.
